# Role of Presenilin in Mitochondrial Oxidative Stress and Neurodegeneration in *Caenorhabditis elegans*

**DOI:** 10.3390/antiox7090111

**Published:** 2018-08-24

**Authors:** Shaarika Sarasija, Kenneth R. Norman

**Affiliations:** Department of Regenerative and Cancer Cell Biology, Albany Medical College, Albany, NY 12208, USA; sarasis@amc.edu

**Keywords:** mitochondria, calcium, *C. elegans*, neurodegeneration, alzheimer’s disease, ROS, presenilin

## Abstract

Neurodegenerative diseases like Alzheimer’s disease (AD) are poised to become a global health crisis, and therefore understanding the mechanisms underlying the pathogenesis is critical for the development of therapeutic strategies. Mutations in genes encoding presenilin (PSEN) occur in most familial Alzheimer’s disease but the role of PSEN in AD is not fully understood. In this review, the potential modes of pathogenesis of AD are discussed, focusing on calcium homeostasis and mitochondrial function. Moreover, research using *Caenorhabditis elegans* to explore the effects of calcium dysregulation due to presenilin mutations on mitochondrial function, oxidative stress and neurodegeneration is explored.

## 1. Introduction

As baby boomers enter retirement age, there could be a potential global health crisis due to the occurrence of various neurodegenerative diseases. Of these, Alzheimer’s disease (AD) poses the greatest threat due to its prevalence, the lack of a clear understanding of its pathogenesis and inefficiency of current therapeutic strategies. AD is the third leading cause of death in the elderly, following heart disease and cancer and is ranked sixth as the cause of death in the general population. Currently, there are about 36 million people suffering from AD and with increased life expectancy, those numbers are expected to double by 2030, and more than triple by 2050 [1].

The brain is highly susceptible to oxidative stress due its increased energy demand and high rates of metabolism, which when left unchecked can result in neurodegeneration. Oxidative stress occurs due to a disparity in redox states brought on by either an excessive generation of reactive oxygen species (ROS) or a reduction in antioxidant function. As the site of ROS production and breakdown, the mitochondria play a critical role in controlling oxidative stress and therefore, mitochondrial dysfunction can be detrimental to organismal health. Thus, understanding the role of ROS in neurodegenerative diseases like AD could provide much needed insight into the development of novel therapeutic targets. In this review, we explore the role of mitochondrial dysfunction and oxidative stress in Alzheimer’s disease.

## 2. Mitochondria: The Story of Energy and ROS Generation

Mitochondria are double membrane bound organelles found in most eukaryotic cells involved in a myriad of functions, including cellular energy generation, signaling and calcium homeostasis. The production of adenosine triphosphate (ATP) in the mitochondria is facilitated by a large number of proteins in the inner membrane using pyruvate generated in the cytosol by the breakdown of glucose. Upon entry into the mitochondrial matrix, pyruvate is decarboxylated to acetyl coenzyme A, which enters the citric acid cycle resulting in the formation of (nicotinamide adenine dinucleotide) NADH, a key electron carrier molecule. These electrons are removed from NADH and passed to oxygen by the enzymes in the electron transport chain (ETC) found in the inner membrane of mitochondria. This process results in the build up of protons in the mitochondrial intermembrane space, and the generation of an electrochemical gradient across the membrane. Protons will flow back into the mitochondrial matrix from the intermembrane space via the proton pore of the ATP synthase, resulting in its rotation, and synthesis of ATP. ROS products like superoxide, peroxide, and hydroxyl radical are a subset of molecules that can be generated as byproducts of the normal metabolism of oxygen. During oxidative phosphorylation, electrons are passed through the various complexes of the ETC via redox reactions, with each acceptor protein complex along the chain having a greater reduction potential than the previous, and with oxygen molecules acting as the final acceptor. While oxygen is normally reduced to produce water, it can get prematurely and incompletely reduced to the superoxide radical (•O^−^_2_) at complexes I and III, which then acts as the precursor to most ROS. ROS can also be generated as a result of exogenous stimuli like ionizing radiation, smoke, pollutants or drugs [2]. Excessive superoxide levels can be detrimental to organismal health and, therefore, cells have protection mechanisms in place to minimize the damage caused by ROS. First, the superoxide dismutase, SOD, will dismutate a superoxide radical into molecular oxygen or hydrogen peroxide, the latter of which can be further processed by catalases and glutathione peroxidases. The production of ATP and, likewise, ROS are affected by intracellular calcium signaling, and, therefore, the maintenance of calcium homeostasis, especially in the endoplasmic reticulum (ER)-mitochondrial region is crucial for optimal cellular health.

## 3. Mitochondria, Calcium and ROS: The Holy Trinity

Various cellular functions including membrane excitability, neurotransmitter release, gene expression, cellular growth, differentiation, free radical species formation and cell death are highly dependent on calcium signaling [3]. Therefore, due to the ubiquitous role of calcium as a second-messenger, cells tightly regulate calcium concentrations [4]. A large electrochemical gradient is created across the ER membrane by the function of the sarco/endoplasmic reticulum calcium ATPase (SERCA) on the ER membrane that pumps calcium from the cytosol into intracellular stores of the ER. Calcium is released from the ER through a variety of mechanisms. For example, G-protein-coupled receptor or receptor tyrosine kinase mediated activation of phospholipase C, and production of inositol-1,4,5-trisphosphate (IP_3_) is necessary for the release of calcium from the ER. The IP_3_ will bind the IP_3_ receptors (IP_3_R) on the ER resulting in release of calcium into the cytosol. The ER-resident calcium-sensitive ryanodine receptors (RYR) will amplify the calcium signals from IP_3_Rs resulting in further calcium release termed calcium-induced calcium release [3].

Upon ER calcium release, a high concentration of calcium is present in close apposition between the ER and mitochondria. In these instances, mitochondria can act as a calcium buffer to stabilize cytosolic calcium levels. Moreover, calcium is a key player in the maintenance of mitochondrial structure and function [5]. In addition to acting as a buffer and inducing morphological changes, calcium entry into mitochondria can affect mitochondrial activity [6,7,8,9,10,11]. For calcium to exert an effect on mitochondrial activity, it needs to cross the mitochondrial outer and inner membranes, and enter into the matrix. Calcium moves into the intermembrane space via the voltage-dependent anion-selective channel (VDAC), a large-diameter (2.53 nm) channel in the outer mitochondrial membrane [12,13]. The uptake of calcium from the intermembrane space into the mitochondrial matrix is then mediated by the mitochondrial calcium uniporter (MCU), an approximately 480-kDa multi-protein complex. The major components of the MCU protein complex include the channel subunit MCU and the calcium-sensing regulatory protein subunit MICU [14,15,16]. MICU modulates MCU function by physically interacting with it and serves as a gatekeeper in MCU-mediated mitochondrial calcium uptake. MICU ensures that mitochondria do not take up calcium when cytoplasmic calcium levels are low [17,18]. Unexpectedly, MCU-null mice do not exhibit any strikingly apparent phenotypes indicative of loss of mitochondrial calcium uptake [19,20]. Also, in MCU-deficient skeletal muscle and MCU-1 null *C. elegans* [21], although decreased, significant levels of calcium were detected in the mitochondrial matrix, which suggests the existence of a compensatory mechanism in vivo. Among other types of mitochondrial calcium uptake transporters proposed [22] are mitochondrial ryanodine receptor (mRYR1), uncoupling proteins (UCP), leucine zipper-EF-hand-containing transmembrane protein 1 (LETM1), mitochondrial calcium current type 2 (mCa2), rapid mode of calcium uptake (RaM), coenzyme Q10, and canonical transient receptor potential channel 3 (TRPC3) [23].

On the other hand, calcium efflux from the mitochondria is predominantly achieved by exchange for Na^+^, which is in turn pumped out of the matrix in exchange for protons [24,25]. However, a supplementary mechanism for calcium efflux exists in the form of the permeability transition pore (PTP). Under pathological conditions where the mitochondrial matrix is overloaded with calcium, calcium binding to the F1 subunit of the ATP synthase, will result in its dissociation from a dimer to monomer, allowing for the formation of the PTP at this junction [26]. PTP could stay in the open state for prolonged periods of time [27]. This prolonged PTP opening will result in the movement of solutes into the mitochondrial matrix, across the concentration gradient, and water following this movement will result in membrane swelling and mitochondrial rupture. Also, PTP opening has been mechanistically linked to cytochrome c release, a key event in apoptosis [28].

A major functional effect of mitochondrial calcium uptake is the stimulation of oxidative phosphorylation [6,7,8,9,10]. Mitochondrial calcium uptake promotes oxidative phosphorylation at multiple steps, including allosteric activation of pyruvate dehydrogenase, α-ketoglutarate dehydrogenase, and isocitrate dehydrogenase [9], as well as stimulation of the ATP synthase (complex V) (40), α-glycerophosphate dehydrogenase [11], and the adenine nucleotide translocase (ANT) [10]. Overall, the increase in mitochondrial calcium concentration results in the synchronized up regulation of the entire oxidative phosphorylation machinery, resulting in elevated respiratory chain activity and increased ATP output. This acceleration of the mitochondrial oxidative phosphorylation machinery can result in an increase in superoxide leak and a concomitant increase in ROS generation, which could result in oxidative damage. Therefore, ER-calcium dysregulation can result in mitochondrial functional changes that could lead to oxidative damage mediated pathologies, like AD.

## 4. Alzheimer’s Disease

AD is the most common cause of dementia in the elderly and is characterized by progressive, irreversible neurodegeneration. Despite its discovery and definition over a century ago, the pathogenesis of this debilitating disease remains a mystery, resulting in the unavailability of successful therapeutic strategies. While the concept of a hereditary/familial mode of AD transmission had been floated since the 1930s [29], it was not until the 1990s that the genetics behind AD was actually delineated. It was noted that majority of these cases of familial inheritance were also associated with an early onset of disease progression and were, thus, classified as early-onset familial Alzheimer’s disease (FAD). Mutations in the gene encoding amyloid precursor protein (APP) were the first to be discovered as a cause of FAD. However, these mutations are responsible for no more than 10–15% of FAD, spurring the search for other FAD associated genes. Genetic linkage studies led to the mapping of a locus to chromosome 14q24.3, which appeared to account for almost 70% of all FAD cases [30,31,32]. A novel gene named *presenilin-1* (*PSEN1*) was identified in this region, whose product resembles an integral membrane protein with multiple transmembrane domains and five missense mutations were identified in this gene that co-segregates with FAD [33]. However, neither *APP* nor *PSEN1* mutations appeared to be the genetic cause of FAD in certain other families including the Volga-German AD families, a group of related families suffering from FAD that descended from one German family [30,31]. A genome-wide search in these FAD families helped identify a locus on chromosome 1q42 whose product showed amino acid sequence homology to PSEN1 and was accordingly named presenilin-2 (PSEN2) [34].

APP is a type I transmembrane protein whose consecutive cleavage by β- and γ-secretases results in the production of amyloid-β (Aβ) peptides [35]. The β-secretase cleavage removes a large part of the ectodomain of APP and generates an APP carboxyl-terminal fragment, which is then cleaved by γ-secretase. Upon γ-secretase cleavage, Aβ is liberated and then found in extracellular fluids such as plasma or cerebrospinal fluid [36]. A large number of FAD-associated APP mutations have been found within and around the Aβ domain [37,38,39] but appear to accelerate disease progression via diverse mechanisms [40,41,42,43,44].

Interestingly, presenilin forms the catalytic core of the multi subunit γ-secretase protease complex, which also contains anterior pharynx-defective 1 (APH-1), presenilin enhancer 2 (PEN-2) and Nicastrin. The γ-secretase complex is assembled inside the early compartments of the ER and transported to other compartments such as the Golgi, lysosomes and the cell surface [45]. While γ-secretase mediates the intramembrane cleavage of over 90 substrates, the PSEN-dependent γ-secretase cleavage necessary for the maturation APP and the Notch receptor, leading to the production of Aβ and the Notch intracellular domain (NICD), respectively, are the best studied [46,47]. There are currently more than 180 FAD-linked PSEN1 mutations identified at 121 sites [48]. The amyloid hypothesis of AD progression postulates that an increase in the ratio of Aβ42 which are highly prone to aggregation to Aβ40 results in their oligomerization and deposition as amyloid plaques and that these plaques can directly cause progressive synaptic and neurite injury or activate microglia and astrocytes, induce inflammation and thereby cause neuronal damage [49,50].

The focus of therapeutic intervention based on the acceptance of the amyloid hypothesis is to prevent disease progression or cause disease regression by targeting the amyloid peptides. Drug therapies to decrease Aβ production include γ-secretase inhibitors, modulators, and β-secretase inhibitors. Aβ aggregation inhibitors, passive and active immunotherapies against Aβ have also been developed as therapeutic measures. However, so far none of these approaches have yielded positive results in clinical trials and despite clearance of amyloid plaques, cognitive decline has not been cogently paused or reversed by these drugs [51,52]. Also, a growing body of evidence suggests that amyloid plaques could be a symptom of the disease rather than the cause of the disease. There is not a high degree of spatial correlation between the presence of amyloid plaques and neurodegeneration, also neurodegeneration is not observed in certain patients with significant amyloid plaque burden and significant amyloid plaques have not been found in some patients suffering from AD [53]. A recent study also showed that 90% of 138 presenilin mutation that are associated with FAD showed reduced production of Aβ40 and Aβ42 [54], casting some doubt on the amyloid hypothesis in AD. Given the evident failure of any drug therapy targeting amyloid peptides, there is a pressing need to explore alternate hypotheses of AD pathogenesis as a means to develop a successful therapeutic intervention.

## 5. Presenilin and the Calcium Hypothesis of Alzheimer’s Disease

Interestingly, a persistent change in calcium homeostasis is a common element between aging and AD, and it was dubbed the “calcium hypothesis of brain aging and Alzheimer’s disease” [55]. This led to work that resulted in the observation that PSEN1-A246E FAD mutation can lead to enhanced IP_3_R-mediated calcium signaling in fibroblasts from asymptomatic FAD patients in 1994 [56]. Remarkably, this calcium dysregulation was detected before the emergence of clinical symptoms of AD and such changes were not present in cells from subjects that failed to develop AD [57]. Similarly, *Xenopus* oocytes expressing mutant PSEN1 and PSEN2 [58] and primary cortical neurons isolated from *PSEN1* knock-in mice display an exaggerated IP_3_R mediated calcium release [59,60]. Also, there are elevated levels of RYR expression in various mouse models of AD; PSEN1-M146V, PSEN2-N141I, 3XTg-AD and TgCRND8, which lead to an increase in calcium release from IP_3_- and caffeine-gated stores in hippocampal and cortical neurons [61,62]. Likewise, another contributor to ER-calcium overload was discovered when increased SERCA activity was observed in *Xenopus* oocytes expressing PSEN1-M146V compared to those with wild-type PSEN1 [63]. While the exact mechanism by which PSEN mutations affect ER-calcium release remains unknown, a possible explanation postulated was that presenilins could be acting as ER-calcium leak channels and the abrogation of the leak channel function as a result of FAD mutations results in overloaded ER calcium stores and exaggerated ER calcium release [64,65]. Strikingly, this was observed to be the case in PS double knockout fibroblasts and in fibroblasts transfected with mutant PSEN1 and PSEN2 constructs [65,66].

**Figure 1:**
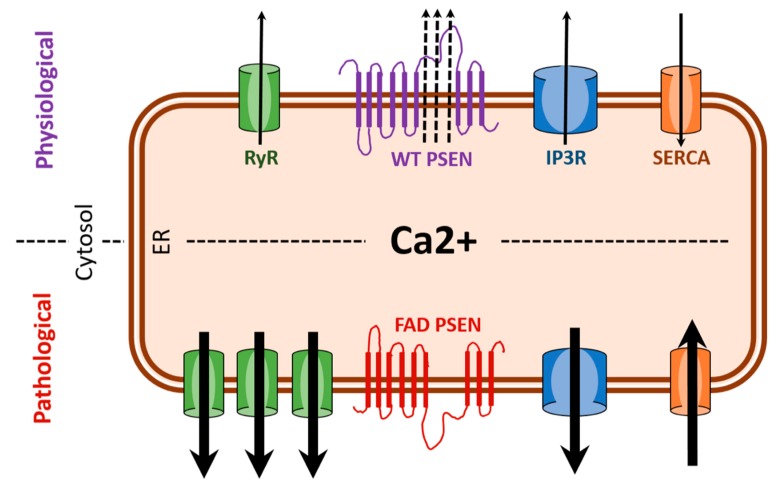
FAD (familial Alzheimer’s disease) presenilin mutations result in enhanced ER (endoplasmic reticulum) calcium release. Under pathological condition associated with FAD, there is excessive ER (calcium release) as a result of overexpression of RYR (ryanodine receptors) and potentiation of IP_3_R (IP_3_ receptors). Also, hyperactivity of SERCA (sarco/endoplasmic reticulum calcium ATPase) pumps and the loss of leak channel function of PSEN (presenilin) can increase ER-calcium stores thereby increasing release of calcium via RYR and IP_3_R. Black arrows indicate direction of calcium movement.

Therefore, we can surmise that FAD mutations in presenilins affect the activity and/or expression of proteins involved in ER calcium signaling and cause enhanced release of calcium from ER stores (Figure 1). Due to the intimate interaction of the ER and the mitochondria upon ER calcium release [67,68], a high concentration of calcium is present in close apposition between the ER and mitochondria causing the mitochondria to act as a calcium buffer to stabilize cytosolic calcium levels and, conversely, calcium is a key player in the maintenance of mitochondrial structure and function [5]. Interestingly, PSENs are subcellularly localized on the ER in regions where ER and mitochondria are in contact, called the mitochondria-associated membranes (MAM) [69]. Also, ER–mitochondrial contacts are increased and ER–mitochondrial crosstalk is enhanced in fibroblasts from FAD and sporadic AD patients, PSEN1 knockout cells, and in cells overexpressing the FAD mutant PSEN2 [70,71,72]. Thus, PSENs bear close witness to ER and mitochondria communication and transfer (e.g., calcium, lipids, ATP) and could play an active role in these functions. It is important therefore to further explore the effect of presenilin mutations on ER calcium signaling, mitochondrial structure and function, and the pivotal role ROS might have in neurodegeneration.

## 6. *C. elegans* as a Model for Alzheimer’s Disease

Presenilins as well as the other components of the γ-secretase complex are an ancient family that are conserved throughout evolution and have been identified in such diverse organisms as plants, amoeba and multicellular animals [73]. Intriguingly, while APP and Notch are well-studied substrates of the γ-secretase, both Notch and APP are not conserved in plants or amoeba. Furthermore, although invertebrates possess Notch and an APP-like molecule, they lack an APP ortholog that contains the Aβ peptide. Moreover, presenilin and the other components of the γ-secretase have been localized to endomembranes [74,75,76] suggesting an ancient role of this protein complex within the cells of diverse organisms and perhaps illuminating the role of presenilin and the γ-secretase in a simple organism will provide insight into AD pathology. Indeed, utilizing the strengths of invertebrate model systems to explore effects of presenilin mutations on calcium homeostasis and mitochondrial function, and the resulting pathology should aid in understanding and treating AD.

*Caenorhabditis elegans* is a simple, free-living, non-parasitic nematode [77] which is a powerful model system that can provide novel insight into the role of presenilin. Adult *C. elegans*, which are predominantly observed as hermaphrodites, can self-fertilize and produce approximately 300 progeny. After hatching, animals go through four distinct larval stages (L1–L4), each punctuated by a molt and they have a relatively short lifespan of ~3 weeks under optimal laboratory conditions. Also, it is relatively simple and inexpensive to culture and maintain *C. elegans* in the laboratory [78]. The lineage of somatic cells in *C. elegans* is largely invariant and the 302 neuron-containing nervous system of adult hermaphrodites has been reconstructed and the connectivity of the entire hermaphrodite nervous system has been mapped [79,80]. Additionally, all cells in the adult soma are post-mitotic, thus similar to human neurons, making them an excellent tool to study neuronal disorders [79,80].

Along with these advantages of *C. elegans* as an animal model, they provide another unique aspect; *C. elegans* do not form amyloid peptides or plaques. The *C. elegans* homolog of APP, APL-1 lacks Aβ peptide sequences and β-secretase recognition sites which renders them incapable of producing amyloid peptides and hence plaques [81,82]. Also, Aβ peptides have never been detected in *C. elegans* [82]. Therefore, it is possible to study the impact of presenilin mutations in *C. elegans* without being confounded by the presence of amyloid plaques.

The *C. elegans* presenilin family encompasses three genes; *hop-1*, *sel-12* and *spe-4* [83,84,85]. Although *hop-1* and *sel-12* are widely expressed, including in muscle and neurons [86], the more distantly related, *spe-4* is exclusively expressed in the male germ line [83]. *sel-12* shows higher sequence identity to human presenilin compared to *hop-1* and has been shown to localize to the endoplasmic reticulum [86]. *sel-12* mutations were initially identified as suppressors that could alleviate developmental defects associated with excessive Notch signaling [84].

## 7. Calcium Homeostasis and Mitochondrial Function Is Disrupted in *C. elegans* Presenilin Mutants

Calcium dysregulation has been observed as a result of presenilin mutations in both in-vitro and in-vivo systems [3] and therefore, the status of calcium homeostasis was examined in *C. elegans sel-12* mutants. Using optogenetic, behavioral and pharmacological assays, it was demonstrated that there is increased ER-calcium signaling in *sel-12* mutants [87] similar to what is observed in vertebrate systems with presenilin mutations [59,61,65,88,89,90]. Calcium homeostasis is crucial for organismal health and, therefore, mechanisms exist to ensure its maintenance. A critical function of mitochondria is to act as a calcium buffer upon calcium release from the ER and increased cytosolic calcium levels. Therefore, given the elevation in ER-calcium release observed in *sel-12* mutants, mitochondrial calcium levels were examined. Strikingly, mitochondrial calcium levels are elevated in both the neurons and body wall muscles of *sel-12* mutants and this phenotype could be suppressed by reducing ER calcium release or mitochondrial calcium uptake [91]. These data are consistent with elevated ER calcium release and, importantly, increased mitochondrial calcium uptake in *sel-12* mutants.

Mitochondria are dynamic organelles that undergo mitochondrial fusion and fission under physiological conditions, however sustained mitochondrial fission can have deleterious effects [92]. Drp1 is a soluble cytosolic protein that mediates mitochondrial fission by assembling into spiral filaments around mitochondrial tubules. These Drp1 spirals will then constrict mitochondrial tubules through conformational changes, driven by GTP hydrolysis resulting in mitochondrial fission [93,94,95]. Mitochondrial morphological changes manifesting as fragmented mitochondria with damaged inner membrane structures have been observed in neurons in AD patients [96] and consistent with this, mitochondrial structural disorganization has been observed in the body wall muscle and neurons of *sel-12* mutants [87], suggestive of elevated Drp1 activity. Interestingly, ER-mediated calcium release and subsequent mitochondrial calcium uptake can impact the activity of Drp1 [93,94,95]. Correspondingly, the higher incidence of structurally disorganized mitochondria in *sel-12* mutants is rescued by reducing ER-calcium release, mitochondrial calcium uptake or knocking down Drp1 [87], suggesting that the loss of *sel-12* results in enhanced ER-mitochondria calcium transfer, which activates Drp1 causing elevated mitochondrial fission.

As previously discussed, elevated ER-calcium release and subsequent mitochondrial calcium uptake can also increase mitochondrial respiration by stimulating various enzymes involved in oxidative phosphorylation. Acceleration of oxidative phosphorylation could result in downstream deleterious effects due to an overproduction of ROS, a product of cellular respiration. Consistent with increased ER-mitochondrial calcium signaling and oxidative phosphorylation, young adult *sel-12* mutants display elevated oxygen consumption rates and increased levels of ROS [91]. Strikingly, similar elevation in oxygen consumption rates and ROS levels were observed in functional astrocytes from induced pluripotent stem cells (iPSCs) derived from AD patients with *PSEN1* mutations suggesting a conserved role for presenilin in mitochondrial respiration and ROS homeostasis [97]. Taken together, these data indicate that presenilin mutations cause increased ER-calcium release, subsequent mitochondrial calcium uptake and concomitant increase in mitochondrial respiration, which results in overproduction of ROS. In contrast to young adult *sel-12* mutants, analyses of oxygen consumption rates in older adult *sel-12* mutants found that mitochondrial respiration was drastically reduced compared to similarly aged wild type animals, indicating that the high level of mitochondrial respiration in young adult *sel-12* mutants cannot be maintained and deteriorates rapidly compared to wild type animals as the mutants age [91].

## 8. Oxidative Stress Mediated Neurodegeneration in *sel-12* Mutants

In neurodegenerative diseases, such as AD, high levels of ROS and defective mitochondrial function have been observed [98]. This is in congruence with the “free radical theory” of aging [99] which suggests that aging and neurodegenerative diseases, could be attributed to the toxic effects free radicals have on various cell constituents. Elevated ER-mitochondria calcium signaling results in enhanced oxidative phosphorylation and this could result in a concomitant increase in ROS levels. Given the role of oxidative stress in neurodegeneration, ROS levels were measured in *sel-12* animals. Markedly, ROS levels were significantly higher in *sel-12* mutants compared to wild type control animals [87]. Moreover, reduction of ER-mitochondrial calcium signaling reduces the levels of ROS observed in *sel-12* mutants [91], indicating that the high ROS production in *sel-12* mutants is caused by increased ER to mitochondria calcium transfer. Similar to the astrocytes from iPSCs derived from FAD patients harboring *PSEN1* lesions [97], fibroblasts isolated from FAD patients harboring different *PSEN1* mutations also showed elevated levels of ROS [91]. Moreover, it was found that blocking mitochondrial calcium uptake in these cells using the mitochondrial calcium uniporter inhibitor, Ru360, could suppress the elevated ROS levels observed in the FAD patient fibroblast. These data suggest a conserved role of presenilin in maintaining normal ER-mitochondrial calcium transfer and, thus, preventing accumulation of ROS.

The impact of elevated ROS levels on the nervous system of *sel-12* mutants was explored in the mechanosensory neurons, a group of six neurons (ALML/R, PLML/R, AVM, and PVM), which perceives light touch to the body of *C. elegans*. As *C. elegans* age, their mechanosensory neurons undergo neurodegeneration characterized by ectopic neurite sprouts, and concurrent inability to perceive mechanosensation by about day 10 of adulthood [100,101,102,103]. Examination of the mechanosensory neurons in *sel-12* mutants, revealed morphological defects such as ectopic neuronal sprouting and axonal breaks, and mechanosensory defects as early as day 1 of adulthood, demonstrating a precocious onset of neurodegeneration. In order to determine whether the neurodegenerative phenotypes observed in these animals were a result of enhanced ER-mitochondrial calcium transfer, ER-calcium release or mitochondrial calcium uptake was inhibited in *sel-12* mutants. This results in a suppression of the neuronal morphology defects associated with neurodegeneration and a normalization of mechanoperception [91].

**Figure 2:**
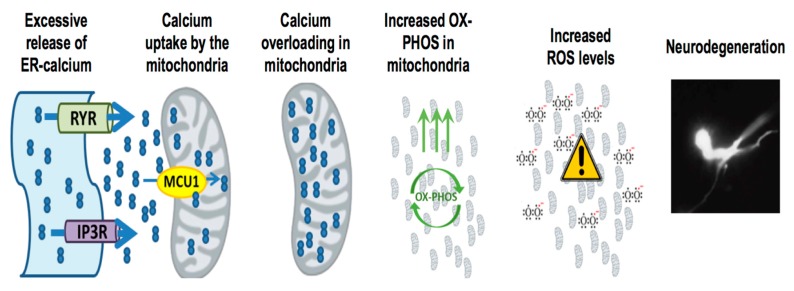
Presenilin mutations result in enhanced ER-mitochondria calcium transfer mediated neurodegeneration. Presenilin mutations result in excessive ER-calcium release, which causes the activation of mitochondrial calcium uniporter MCU-1 (mitochondrial calcium uniporter 1) and subsequent uptake of calcium into the mitochondria. With increased calcium uptake into the mitochondria, it stimulates mitochondrial respiration and increases ROS (reactive oxygen species) generation, resulting in neurodegeneration.

Given that reducing ER-calcium release and mitochondrial calcium uptake can attenuate neurodegeneration and lower ROS levels in the *sel-12* mutants, the role of elevated ROS levels in causing neurodegeneration was investigated. *sel-12* mutants were treated with a mitochondrially-targeted antioxidant, (2-(2,2,6,6-Tetramethylpiperidin-1-oxyl-4-ylamino)-2-oxoethyl) triphenylphosphonium chloride (MitoTEMPO). Treatment with MitoTEMPO resulted in the restoration of normal neuronal structure and function in these animals [91], indicating that mitochondrially generated ROS is causing neurodegeneration in the *sel-12* mutants. Taken together, these data demonstrate that SEL-12/presenilin function is required to maintain normal calcium transfer from the ER to the mitochondria and in the absence of optimal SEL-12/presenilin function elevated calcium is transferred from the ER to the mitochondria increasing oxidative phosphorylation and ROS levels leading to neurodegeneration (Figure 2). Moreover, these data indicate that while increased oxidative phosphorylation can lead to increased ATP production, it is the elevated ROS that causes cellular damage.

## 9. Presenilin and γ-Secretase Function

Presenilin is the catalytic core of the γ-secretase and mutations in presenilin that are associated with FAD are thought to arise because they promote the production of the aggregation prone Aβ42 [104]. However, several studies have implicated that many of the FAD mutations actually abolish γ-secretase activity. Indeed, a recent study employed a knock-in approach to investigate the effects two FAD *PSEN1* mutations have on γ-secretase function in mice and found that these mutations abolished γ-secretase activity [105]. Likewise, another recent study investigated 138 distinct FAD *PSEN1* mutations and found that 90% of these mutants decreased Aβ production [54]. Yet 10% of these mutations showed normal or elevated Aβ production. Thus, it is unclear how *PSEN1* mutations are causing neurodegeneration in affected patients. In *C. elegans* a major function of presenilin is in promoting Notch signaling. Indeed, loss of *sel-12* leads to defects in egg laying behavior due to loss of Notch signaling [106]. To determine if Notch or γ-secretase activity has a role in mitochondrial or neurodegenerative phenotypes observed in *sel-12* mutants, several approaches were taken. First, analyses of *C. elegans* Notch mutants did not reveal any mitochondrial or touch behavioral defects as observed in *sel-12* mutants [87,91], suggesting Notch does not have a role in the mitochondrial dysfunction or neurodegeneration observed in *sel-12* mutants. Moreover, γ-secretase activity was investigated by both pharmacological inhibition and the generation of a protease dead SEL-12 protein. Both of these approaches found that mitochondria and neuronal activity were normal [87,91], demonstrating that the role *sel-12* has in regulating ER-mitochondrial calcium signaling and preventing neurodegeneration is independent of its γ-secretase activity.

## 10. Studying Aβ Toxicity in *C. elegans*

The effects of Aβ have been studied in *C. elegans* by generating transgenic strains that overexpress human Aβ42 in all neurons, a subset of neurons or in the body wall muscles. *C. elegans* expressing Aβ42 in the body wall muscle show age-dependent paralysis [107,108], which progresses even faster when animals were raised at 25C [108] and expression of Aβ42 in the glutamatergic neurons results in pervasive neurodegeneration consistent with the neurotoxic effects of Aβ [109]. While it is clear that ectopic overexpression of Aβ42 in *C. elegans* causes cellular dysfunction, it is unclear whether this dysfunction phenocopies the defects that arise in *sel-12* mutants. Interestingly, previous studies of animals overexpressing Aβ42 in the body wall muscle or pan-neuronally have shown a reduction of mitochondrial activity [110,111], which is reminiscent of mid-late life adult *sel-12* mutants [91]. Although animals expressing Aβ42 pan-neuronally show defects in touch behavior similar to *sel-12* mutants, none of the mitochondrial defects or axonal abnormalities associated with *sel-12* mutants are observed [91], suggesting loss of *sel-12* leads to neuronal degeneration via a distinct mechanism from ectopic overexpression of Aβ42.

## 11. Future Directions and Conclusions

Thus far, research into treatments for AD has relied heavily on the amyloid hypothesis, which posits that the toxicity of the Aβ peptides and its aggregation to form amyloid plaques drives AD pathogenesis. While it is clear that Aβ accumulation is toxic, the repeated failures in late stage clinical trials of anti-Aβ therapies like semagacestat, a γ-secretase inhibitor, [51] and solanezumab, a monoclonal antibody targeting amyloid plaques [52] highlight the lack of understanding behind the exact role of Aβ peptides and importantly the cause of AD. This gives further support to the urgency in exploring other non-amyloid hypotheses of Alzheimer’s disease pathogenesis.

Calcium homeostasis is critical to normal cellular health and function and its dysregulation, especially enhanced ER-mitochondrial calcium signaling, is observed in various systems modeling AD. Mutant presenilin mediated increase in ER calcium release and subsequent mitochondrial calcium uptake can result in the acceleration of the oxidative phosphorylation machinery, resulting in increased ROS production and consequent oxidative stress as seen in *C. elegans* with presenilin mutations and in human cells derived from FAD patients with PSEN1 mutations [91,97]. Oxidative stress is detrimental to organismal health and can cause cellular damage, especially in tissues with high metabolic needs like the nervous system, and if left unchecked can result in neurodegeneration. Importantly, oxidative stress has been implicated in AD and likely plays a key role in its pathology [112,113] and may precede or even promote protein aggregation (e.g., amyloid plaques and neurofibrillary tangles).

Currently the only therapeutic options available to patients are drugs that help control the symptoms of the disease. However, treating just the symptoms of this disease is not sufficient any longer, especially with the expected doubling in patient numbers by 2030 as baby boomers enter retirement age. While the presence of Aβ and its neurotoxic effects are well characterized, it is clear that they are not the sole culprits and targeting Aβ does not appear to be an effective therapeutic strategy. However, it is possible that ER-mitochondrial calcium dysregulation and associated oxidative stress could be creating an environment that facilitates aggregation. Taken together, this gives credence to a non-amyloid, calcium-mitochondria-ROS dependent mode of Alzheimer’s disease pathogenesis and highlights the need to explore alternative therapeutic targets for Alzheimer’s disease.
